# *FGF20* Secreted From Dermal Papilla Cells Regulate the Proliferation and Differentiation of Hair Follicle Stem Cells in Fine‐Wool Sheep

**DOI:** 10.1111/jpn.14081

**Published:** 2024-12-20

**Authors:** Yali Song, Yuhang Li, Zengkui Lu, Lin Yue, Tong Xiao, Bohui Yang, Jianbin Liu, Chao Yuan, Tingting Guo

**Affiliations:** ^1^ Lanzhou Institute of Husbandry and Pharmaceutical Sciences, Chinese Academy of Agricultural Sciences Lanzhou China; ^2^ College of Animal Science and Technology Ningxia University Yinchuan China; ^3^ Sheep Breeding Engineering Technology Research Center of Chinese Academy of Agricultural Sciences Lanzhou China; ^4^ Key Laboratory of Animal Genetics and Breeding on Tibetan Plateau Ministry of Agriculture and Rural Affairs Lanzhou China

**Keywords:** co‐culture, dermal papilla cells, *FGF20*, fine‐wool sheep, hair follicle stem cells

## Abstract

Wool traits determine the market value of fine‐wool sheep, and wool fibre‐breaking elongation (fibres can be stretched or elongated before they break) is one of the important wool traits. The interaction between hair follicle stem cells (HFSCs) and dermal papilla cells (DPCs) determines hair follicle development in fine wool sheep, thereby directly influencing wool traits. A genome‐wide association study based on pre‐sequencing data identified *FGF20*, which was significantly associated with wool fibre‐breaking elongation. The study reveals that the regulatory mechanism of *FGF20* secreted from DPCs affects the proliferation and differentiation of HFSCs through a co‐culture system, to provide a new perspective for fine‐wool sheep breeding. After knocking down *FGF20* expression in DPCs, the results showed that the expression of *fibroblast growth factor receptor 2* (*FGFR2*) and *fibroblast growth factor receptor 3* (*FGFR3*) in DPCs and HFSCs was significantly decreased (*p* < 0.05), the number of EdU‐positive cells and cell viability was significantly decreased (*p* < 0.01), and the apoptosis rate was significantly increased (*p* < 0.05). Meanwhile, the differentiation markers of *SOX9*, *NOTCH1* and *β‐Catenin* in HFSCs were also significantly reduced (*p* < 0.05). These findings indicate that *FGF20*‐knockdown in DPCs of fine‐wool sheep inhibits the proliferation and differentiation of HFSCs in the co‐culture system, providing a theoretical basis for elucidating the regulatory mechanism of hair follicle self‐renewal and differentiation of fine‐wool sheep and providing a co‐culture system for regenerative medicine.

## 1 Introduction

The skin serves numerous vital functions in mammals, including safeguarding the body, producing sebum, sweat, and pheromones, as well as regulating body temperature (Nguyen and Soulika 2019). The hair follicle is a diminutive multicellular organ located within the skin, and its morphogenesis commences during the embryonic period. The growth process of hair follicles is governed by a multitude of factors. With the changing of seasons, wool follicles exhibit the characteristic of periodic cycling, which dictates the growth and shedding of wool. They are primarily categorized into three stages: growth, degeneration and resting (Schneider, Schmidt‐Ullrich, and Paus 2009). The hair follicle comprises over 20 distinct types of cells, including hair matrix cells, dermal papilla cells (DPCs), and hair follicle stem cells (HFSCs), among others. The hair follicle acts as the signalling centre, wherein DPCs engage in the regulation of the hair follicle's periodic growth through a myriad of growth factors and signalling pathways (Topouzi et al. 2017). HFSCs are the multipotent stem cells, which are important seed cells for tissue reconstruction of hair follicles. When the body experiences external stimuli and sustains skin damage, HFSCs undergo rapid proliferation and exhibit multidirectional differentiation potential. Reports indicate that HFSCs can differentiate into various functional cell types, including adipocytes, cardiomyocytes and osteoblasts (Yamane et al. 2021). The periodic cycling of hair follicles is driven by HFSCs. At the conclusion of the telogen phase, the activation of HFSCs induces hair follicles to re‐enter the growth mode, thereby initiating the subsequent hair follicle cycle. Consequently, HFSCs play a vital role in the entirety of the hair follicle growth cycle (Ji et al. 2021). DPCs can serve as a pivotal cellular model for research on hair follicle development, while HFSCs possess considerable potential for the treatment of various diseases. During the growth cycle of hair follicles, the interaction, proliferation and signal exchange between DPCs and HFSCs are essential for initiating hair follicle regeneration (Rahmani et al. 2014). Thus, establishing a co‐culture model of DPCs and HFSCs holds great significance.

The *fibroblast growth factor* (*FGF*) family is extensively expressed in both humans and animals, comprising 22 members (Xie et al. 2020). The family plays a crucial role in various biological processes, including organogenesis, angiogenesis, cell migration, and differentiation (Beenken and Mohammadi 2009). While research on *FGF20* has predominantly centred on Parkinson's disease and various tumour types, investigations pertaining to hair follicle growth and development remain limited. *FGF20* is a quintessential target gene within the Wnt signalling cascade (Yang, Huh, and Ornitz 2018). Xu et al. (2018) discovered that recombinant human *FGF20* stimulates the proliferation and differentiation of hair stromal cells by activating the Wnt/β‐catenin signalling pathway, thereby facilitating hair growth. Huh et al. (2013) found that *FGF20* plays a role in the activation of dermal condensation, facilitating the formation of DPC precursors and inducing hair follicle morphogenesis. The authors previously screened for *FGF20* expressed in DPCs and its involvement in hair follicle morphogenesis in fine‐wool sheep through a combination of long noncoding RNA and coding RNA analyses (Yue et al. 2016). Nevertheless, the mechanism by which *FGF20* mediates DPC‐induced proliferation and differentiation of HFSC remains to be further clarified.

Wool traits, including wool fibre diameter, diameter variation coefficient and wool fibre breaking elongation, are closely related to the periodic development of hair follicles. Among them, the elongation at break of wool serves as a significant quality index, reflecting the tensile properties and durability of fabrics. In the textile industry, it is generally observed that a greater elongation at break corresponds to a more relaxed state of the yarn within the fabric; this results in reduced internal stress, an increased shrinkage rate and a lower interweaving rate, which in turn enhances the curvature of the yarn. Furthermore, a more rounded cross‐section contributes to a fabric that is more vibrant, rich and elastic. Although it is frequently subjected to stretching, it exhibits remarkable resilience against breakage, rendering it both comfortable to wear and durable. Genome‐wide association study (GWAS) is a method for identifying candidate genes for livestock economic traits. Using it, we can also screen target genes associated with phenotypic traits in the genome as target genes for molecular design breeding. However, these target genes urgently need further in‐depth functional validation at the molecular‐cellular level. GWAS on growth traits using WGS data has revealed that some single‐nucleotide polymorphism (SNPs) and candidate genes are related to the body weight of Chinese fine‐wool sheep (Lu et al. 2020). In this study, we screened a candidate gene, FGF20, associated with the wool fibre‐breaking elongation trait of fine‐wool sheep through GWAS using the pre‐obtained resequencing data and subsequently validated its functions. DPCs and HFSCs were isolated and cultured, followed by their co‐culture to maximally simulate the microenvironment and their interactions in vitro. We investigated the mechanism by which *FGF20*‐mediated DPC‐induced proliferation and differentiation of HFSCs at both cellular and molecular levels, thereby offering novel insights and methodologies for the artificial breeding of superior fine‐wool sheep.

## 2 Materials and Methods

### 2.1 Animals and Sample Preparation

Blood samples were collected from a total of 460 healthy adult fine‐wool sheep for resequencing in the context of GWAS analysis (see Lu et al. (2020) for details). For each animal, the mid‐side wool sample (weight: 200 g) was obtained from the shoulder blades using shears and subsequently sent to the National Animal and Fur Quality Supervision and Inspection Center in the Rural Ministry (Lanzhou, China). The inspection process standards were implemented in accordance with the relevant national standards (GB/T 27629‐2011). The wool fibre‐breaking elongation trait was measured on the local ranch.

Three healthy 3‐month‐old Alpine Merino sheep, all feeding the same diet, were selected from the Gansu Provincial Sheep Breeding Technology Extension Station (Zhangye, China). Under sterile conditions, a skin sample measuring approximately 1 cm^2^ was collected by Acu‐Punch Skin Biopsy Punch (10 mm, USA) from the upper edge of the scapula of a fine‐wool sheep. The samples were washed with 75% alcohol for 15 s and then with phosphate‐buffered saline (PBS) containing 1% double antibody three times. Subsequently, they were placed in DMEM/F12 medium containing 2% double antibody and were brought back to the laboratory for subsequent cell culture.

### 2.2 Genome‐Wide Association Study

Blood sample DNA was extracted by the traditional phenol‐chloroform method, and genome resequencing was performed on the Illumina HiSeq X platform. The clean reads generated from the sequencing were aligned to the sheep reference genome (*Oar 4.0*) (Li and Durbin 2010). SAMtolls software was used to detect high‐quality SNPs (coverage depth > 3, proportion of mis‐assignments < 10%, and minor allele frequency > 5%). A Bonferroni correction was subsequently applied (Li et al. 2009). These raw resequencing data were downloaded from previously published studies (https://dataview.ncbi.nlm.nih.gov/object/PRJNA1162637) (Lu et al. 2020).

The correlations between the SNPs and the wool fibre breaking elongation were analyzed using a mixed linear model in EMMAX software. Population genetic structure and sex were regarded as fixed effects, and individual genetic kinship was regarded as random effects to correct the influence of population structure and individual genetic kinship. The statistical analysis model used in this study was *y* = X*а* + Z*β* + W*μ* + *e*, where *y* represents the phenotypic trait, X is a matrix of fixed effects, *а* is the estimation parameter of the fixed effects, Z is a matrix of SNPs, *β* is the effect of the SNPs, W is a matrix of random effects, *μ* is the predicted random individuals and *e* is the random error, with the distribution *e*~ (0, *δ_e_* ^2^). The significance threshold for the GWAS was established using the Bonferroni correction method. The total Type‐I error rate was controlled at 5% and the significance threshold for the genome was 0.05/Nsnp, where Nsnp is the number of SNPs remaining after quality control. The Manhattan plot and Quantile–Quantile (QQ) plot were obtained from the correlation results.

### 2.3 Isolation, Purification and Identification of Primary DPCs and HFSCs

Under sterile conditions, the connective tissue was meticulously excised from the skin samples. The epidermis and dermis were separated by digestion in 2 mg/mL dispase, and hair follicles were pulled out. To isolate DPCs, the dermal papillae at the lower part of the extruded hair follicles were placed in a six‐well plate and digested with 0.25% trypsin. When a large number of cells in the culture dish migrated from the base in the hair bulb to the area of invagination, half of the medium was replaced. To isolate HFSCs, the hair follicles were directly digested in 0.25% trypsin and observed under a microscope. After approximately 30 min, the digestion was terminated when the cells were free. Subsequently, DPCs and HFSCs were purified by trypsin according to the differential adhesion degrees of DPCs and HFSCs. After three generations of purification, more than 95% pure DPCs and HFSCs were obtained. Cellular immunofluorescence was used to identify the DPCs by labelling them with the proteins α‐SMA and SOX2, and the HFSCs by labelling them with the proteins CK14 and CK19. The fourth‐ or fifth‐generation (P_4_ or P_5_) DPCs and HFSCs were individually inoculated onto a 12‐well plate. Upon reaching 80%–90% confluence, the medium was discarded, and the cells were washed three times with PBS. The cells were then fixed in 4% paraformaldehyde at room temperature for 20 min and subsequently washed three times with PBS. Then they were incubated in 0.5% Triton X‐100 for 10 min, washed three times with PBS, and blocked in 3% BSA for 1 h. Next, the cells were washed three times with PBS again and incubated overnight at 4°C with a 1:100 diluted primary antibody specific to the labelling protein. Similarly, the cells were washed three times with PBS and incubated for 1 h at room temperature with a 1:100 dilution of Cy3‐labelled goat anti‐rabbit secondary antibody. Finally, the cells were washed three times with PBS, incubated with DAPI at room temperature for 10 min, and then imaged using a confocal microscope. The cells were cultured in the growth medium shown in Table 1 at 37°C in a 5% CO_2_‐saturated humidity incubator.

**Table 1:** Cell culture medium preparation reagents.

	DPCs	HFSCs
DMEM/F12 medium	√	√
5 µg/mL insulin	√	√
10 µg/mL epidermal growth factor	√	√
0.5 µg/mL hydrocortisone		√
10% FBS	√	√

### 2.4 Transfection of FGF20‐siRNA and Detection of Efficiency

The transfection efficiency of *FGF20* siRNA was detected before the establishment of co‐culture cell models. The fourth‐ or fifth‐generation DPCs were inoculated into the lower chamber. Upon reaching 60%–70% confluence, FGF20‐siRNA‐1098, FGF20‐siRNA‐773, FGF20‐siRNA‐900, FGF20‐siRNA‐1006, siRNA‐NC or FAM‐labelled NC (designed and synthesized by Gemma Gene Company, with the sequence shown in Table 2) were transfected into DPCs using transfection reagents (Zeta Life, USA) in accordance with the manufacturer's instructions (transfection reagent:siRNA = 1:1). The efficiency of RNA interference was evaluated using qRT‐PCR, and the optimal FGF20‐siRNA was screened for subsequent cell co‐culture experiments.

**Table 2:** RNA oligonucleotide synthetic sequence.

Gene name	Sequence (5′‐3′)
siRNA‐FGF20‐773	S: ACAGCCUCUUCGGUAUCCUTT
	AS: AGGAUACCGAAGAGGCUGUTT
siRNA‐FGF20‐900	S: CUGAAUGCAUCUUUAGAGATT
	AS: UCUCUAAAGAUGCAUUCAGTT
siRNA‐FGF20‐1006	S: CAAAGAUGGAACUCCAAGATT
	AS: UCUUGGAGUUCCAUCUUUGTT
siRNA‐FGF20‐1098	S: CUGAACUGUAUAAGGACCUTT
	AS: AGGUCCUUAUACAGUUCAGTT

### 2.5 Construction of DPC and HFSC Co‐Culture Model

**Figure 1:**
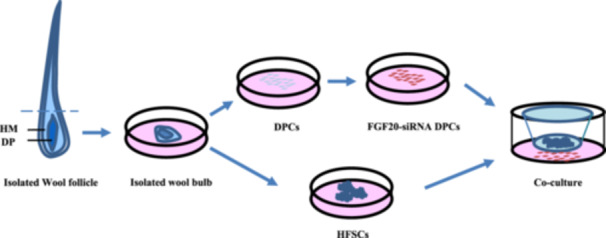
A schematic model of co‐culture. DPCs and HFSCs were co‐cultured using transwell inserts with a 0.4‐μm porous membrane for separation. Each cell type was grown independently on the transwell plates. DP, dermal papilla; HM, hair matrix. [Color figure can be viewed at wileyonlinelibrary.com]

The co‐culture model of DPCs and HFSCs was established using a transwell cell chamber with a 0.4‐μm pore size. The fourth‐ or fifth‐generation DPCs were inoculated into the lower chamber. Upon reaching 60%–70% confluence, siRNA‐FGF20 and siRNA‐NC were transfected into DPCs using transfection reagents. After 24 h of transfection, the fourth‐ or fifth‐generation HFSCs were seeded into the upper chamber and co‐cultured for 24 h to perform subsequent cell experiments. A part of the cells was collected for RNA extraction and related mRNA detection (Figure 1).

### 2.6 Total RNA Extraction and qRT‐PCR

Cells from the co‐culture model of DPCs and HFSCs were collected in TRIzol for the extraction of total RNA. The concentration and quality of the RNA were assessed using an ultraviolet microspectrophotometer and electrophoresis, respectively. qRT‐PCR primers for *FGF20*, its receptors *FGFR2* and *FGFR3*, as well as cell differentiation and signalling pathways, such as *SOX9*, *NOTCH1* and *β‐Catenin*, were designed based on the mRNA sequences of the candidate genes published in the NCBI (GenBank). These primers were generated using Primer Premier 5.0 and synthesized by Xi'an Kinko Jersey Bio‐Tech Company (Table 3). A PrimeScript RT Reagent Kit with gDNA Eraser (Vazyme, Nanjing, China) was employed for cDNA synthesis. Employing the cDNA as a template, the expression of *FGF20* and related mRNAs in the cells were measured with ChamQ Universal SYBR qPCR Master Mix (Vazyme, Nanjing, China) on a CFX96 Touch Real‐Time PCR Detection System (Bio‐Rad Laboratories Inc, Hercules, CA, USA). The following components were used for the qRT‐PCR system (20 μL total volume): 10 µL qPCR mix, 0.8 µL upstream and downstream primers, 2 µL cDNA (100 ng), and filled with ddH_2_O. The PCR protocol consisted of pre‐denaturation at 94°C for 30 s, denaturation at 94°C for 5 s, and extension at 60°C for 30 s (40 cycles). The cDNA template was normalized using *β‐actin* (*ACTB*) as the internal reference gene.

**Table 3:** qRT‐PCR primers.

Gene name	Prime sequence (5′‐3′)	Product length (bp)	Annealing temperature (°C)
*FGF20*	F: CCACAGCCTCTTCGGTATCCT	282	60
	R: TGGTGTCTTTTGGACCTGGC		
*FGFR2*	F: AGAGTGATGTCTGGTCCTTCG	328	56
	R: GAAGAGCAAGAACTCCTGGTG		
*FGFR3*	F: GCTCAAGCGACAGGTAACAGT	302	59
	R: TGTCCGTGGCGTCATCTTT		
*SOX9*	F: CGAAACTGGACTGGAAACCT	113	60
	R: GTTCTCTCTGCCTGTTTGGA		
*NOTCH1*	F: ACAACGCCTACCTCTGCTTC	161	60
	R: ACACATGCTCCCTGTGTAGC		
*β‐Catenin*	F: ACACAGTTCGATGCTGCTCA	126	59
	R: GATTGCACGTGTGGCAAGTT		
*ACTB*	F: GGCATCCTGACCCTCAAGTA	203	57
	R: GGGGTGTTGAAGGTCTCAAA		

### 2.7 Assessment of Proliferation and Viability in the DPC and HFSC Co‐Culture Model

After 24 h of co‐culture, EdU staining was performed using the EdU kit (Biyuntian, China). The EdU stock solution was diluted with cell culture medium at a ratio of 1:500 to prepare a 2× EdU working solution, which was added to the co‐culture cell plate at a ratio of 1:1 and incubated for 2 h. The culture medium was discarded, and 1 mL of fixative (4% paraformaldehyde) was added. The cells were then fixed at room temperature for 15 min. Following fixation, 2.5 mL/well of the washing solution (PBS containing 3% BSA) was added, and the cells were washed three times, with each wash lasting for 5 min. Then, 2.5 mL/well of the permeabilization solution (containing 0.3% Triton X‐100 PBS) was added, and the cells were incubated at room temperature for 15 min and then washed two times. After the washing solution was discarded, 500 μL/well of click reaction solution was added, and the cells were incubated at room temperature in the dark for 30 min. Following this incubation, the cells were washed three times, with each wash lasting 5 min. Subsequently, 500 μL of Hoechst 33342 was added to each well, and the cells were incubated at room temperature for 10 min in the dark. Finally, the cells were washed three times. Fluorescence was observed and photographed using an inverted fluorescence microscope. The CCK‐8 kit was used to detect the cell viability of co‐cultured DPCs and HFSCs. Specifically, 100 μL of CCK solution was added to the upper chamber, while 200 μL was added to the lower chambers. After a 2‐h incubation period, the absorbance at 450 nm was measured using a microplate reader. These experiments were conducted in triplicate to ensure biological replicability.

### 2.8 Flow Cytometric Analysis of Apoptosis

Apoptosis was detected by flow cytometry. The Annexin V‐FITC apoptosis detection kit (Biyuntian, China) was used for detection in accordance with the manufacturer's instructions. Fourth‐ or fifth‐generation DPCs were seeded in six‐well plates and transfected with either FGF20‐siRNA or NC siRNA. Following 24 h incubation, fourth‐ or fifth‐generation HFSCs were seeded in the upper chamber. After an additional 24 h of culture, the cell culture medium was aspirated into a centrifuge tube, and the adherent cells were washed once with PBS. Subsequently, 800 μL of trypsin was added to digest the cells. Following digestion, the cell culture medium in the centrifuge tube was added back, and the cells were gently pipetted. The cells were then transferred to a centrifuge tube and centrifuged at 1000 × *g* for 5 min. Thereafter, the supernatant was discarded, and the cells were resuspended in PBS. The resuspended cells were centrifuged at 1000 × *g* for 5 min. The supernatant was discarded, and 195 µL of Annexin V‐FITC binding solution was added to the cells. Then, 5 µL of Annexin V‐FITC and 10 µL of propidium iodide staining solution were gently mixed and applied to the cells, which were then incubated at room temperature in the dark for 15 min before being placed in an ice bath. Detection was performed using a flow cytometer within 1 h. The experiment was repeated three times.

### 2.9 Statistical Analysis

The 2^−ΔΔC*t*^ method was used to analyze quantitative fluorescence results. The data were expressed as х ± s. The single factor analysis of variance by SPSS 22.0 software was used for statistical analysis. *p* < 0.05 was defined as statistically significant.

## 3 Results

### 3.1 Genome‐Wide Association Study

**Figure 2:**
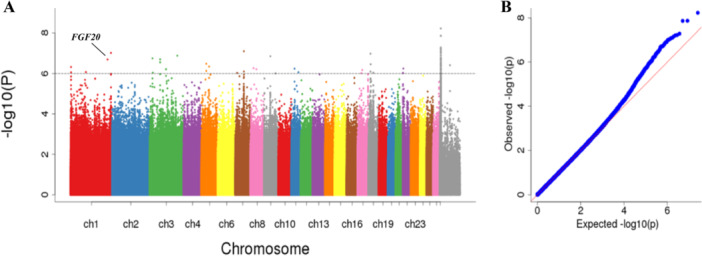
Manhattan (A) and QQ plots (B) of the wool fibre breaking elongation trait for fine‐wool sheep breeds. The grey horizontal lines in the Manhattan plots indicate the suggestive significance (10^−6^) thresholds. The blue dots in the QQ diagram represent the −log^10^ (*p* value) of the entire study, and the red line represents the expected values under the null hypothesis of no association. [Color figure can be viewed at wileyonlinelibrary.com]

Following sequencing conducted on the Illumina HiSeq Xten platform, high‐quality next‐generation sequencing data from the 460 fine‐wool sheep were acquired. The QC results (Q20 ≥ 96.19% and Q30 ≤ 90.80%) indicated high sequencing quality. Subsequent to quality control, a total of 12,725,769 high‐quality SNPs were identified. Over 98.54% of the clean reads were successfully aligned to the sheep reference genome using BWA software. The coverage depth of each sample after genome alignment was approximately 9.43 times. Before performing the GWAS analysis, the population structure of the test population had to be analyzed and corrected accordingly. The effect of population stratification on phenotypic variation needs to be corrected when performing association analysis. For the wool fibre breaking elongation trait, 31 significantly correlated SNPs exceeding the threshold line were detected on Chr1, Chr3, Chr5, Chr7, Chr8, Chr9, Chr11, Chr17, Chr18 and Chr 22, and were effectively annotated to 11 genes. The significantly related SNPs, which were significantly associated with the wool fibre‐breaking elongation trait, were detected on chromosome 1 and annotated to the *FGF20* gene (Figure 2A,B). Previous studies have found that *FGF20* is expressed in DPCs and is involved in hair follicle morphogenesis in fine‐wool sheep (Yue et al. 2016). Meanwhile, *FGF20* is involved in the activation of dermal condensation to form DPC precursors and to induce hair follicle morphogenesis. Therefore, this gene was selected for further gene function validation in this study.

### 3.2 The Morphology and Identification of the Primary DPCs and HFSCs

**Figure 3:**
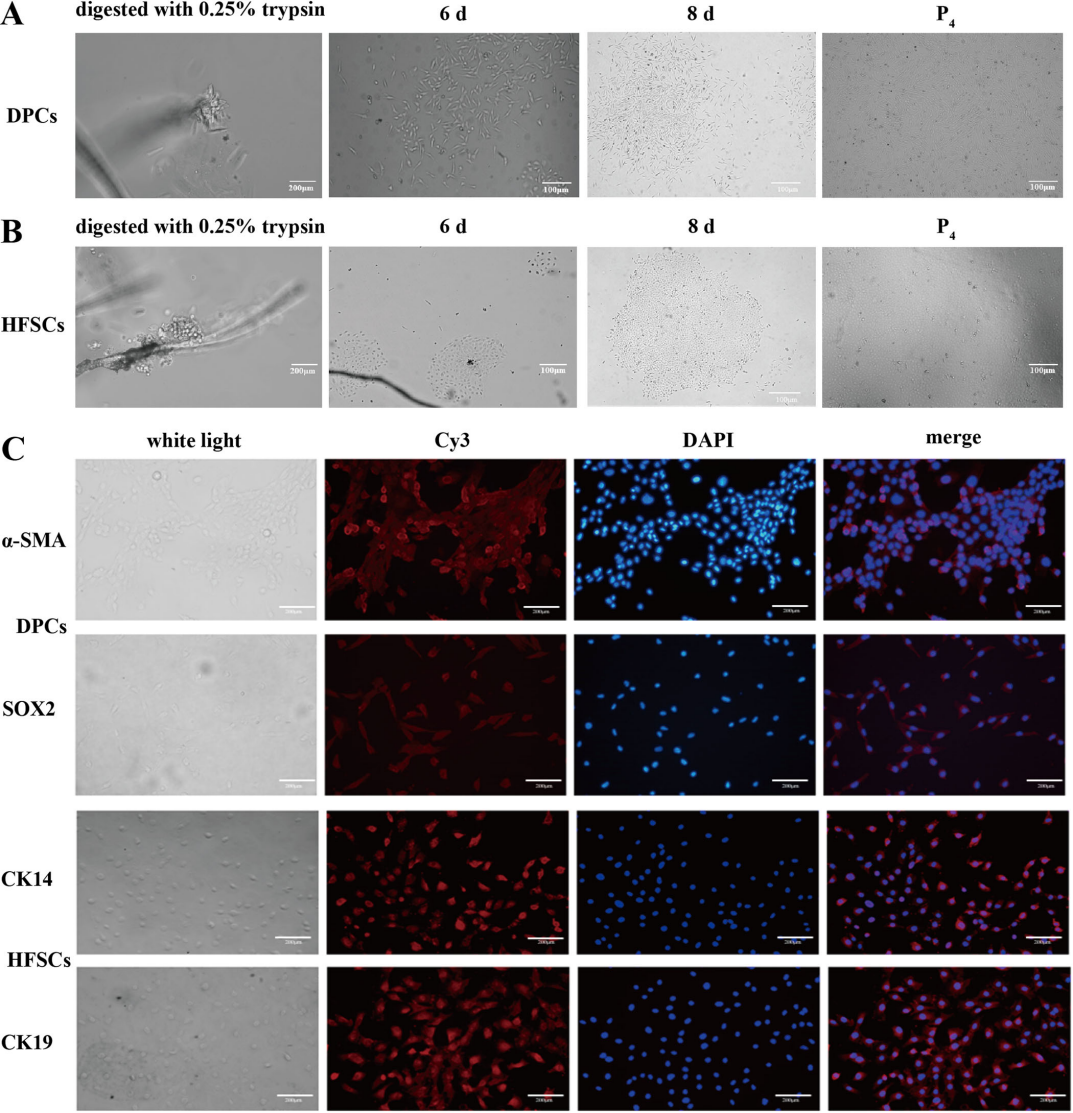
Characterization of primary DPCs and HFSCs. (A) Morphology of primary fine‐wool sheep DPCs at trypsin digestion, 6th, 7th day and 4th passage after purification. (B) Morphology of primary fine‐wool sheep HFSCs at trypsin digestion, 6th, 7th day and 4th passage after purification. (C) Immunofluorescence analysis of isolated DPCs and HFSCs using antibodies corresponding to DPCs and HFSCs specific marker α‐SMA, SOX2, CK14 and CK19. α‐SMA, SOX2, CK14 and CK19 are indicated with red fluorescence. [Color figure can be viewed at wileyonlinelibrary.com]

Under sterile conditions, primary DPCs were isolated from fine‐wool sheep through enzymatic digestion and mechanical separation (Figure 3A). When observed under an inverted microscope, hair follicle tissue was digested using 0.25% trypsin, and the cells were obtained. When cultured to the 6th day, the cells adhered to the wall, with triangular or polygonal cell morphology and a relatively large cell body. On the 7th day, it was found that most of the cells grew radially around and formed a dense area.

HFSCs were isolated from fine‐wool sheep by enzymatic digestion and mechanical separation under sterile conditions (Figure 3B). When observed under an inverted microscope, cell migration during separation was observed in 0.25% trypsin digestion. After 6‐day culture, the cells adhered to the wall and showed more aggregated growth of cell clumps. On the 8th day, it was found that the cells proliferated rapidly and their cell morphology was flat.

HFSCs and DPCs were identified by immunofluorescence (Figure 3C). The fourth‐ or fifth‐generation DPCs (Figure 3B) were assessed for hepatocyte‐specific markers α‐SMA and SOX2 using corresponding antibodies. Results showed that α‐SMA and SOX2 were expressed in DPCs. Moreover, CK14 and CK19 were expressed in the fourth‐ or fifth‐generation HFSCs (Figure 3B). Therefore, the purity of the isolated DPCs and HFSCs was high and suitable for subsequent research.

### 3.3 Detection of the Transfection Efficiency of FGF20‐siRNA in DPCs

**Figure 4:**
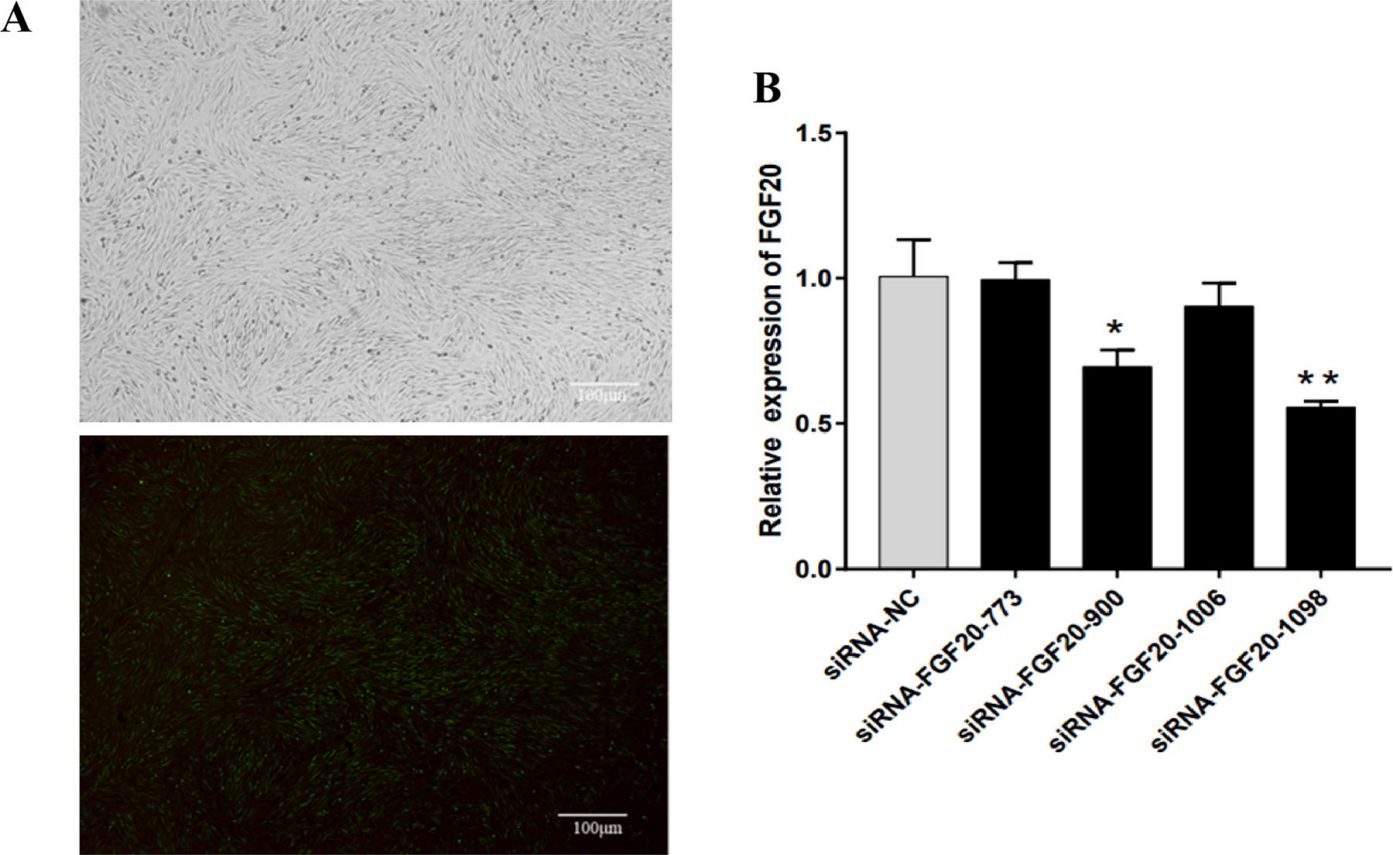
Detection of siRNA transfection efficiency in DPCs. (A) The transfection efficiency of FGF20‐siRNA in DPCs was observed under an inverted fluorescence microscope. (B) The interference efficiency of FGF20‐siRNA was detected by qRT‐PCR. ** indicates *p* < 0.01, * indicates *p* < 0.05.

To investigate the effect of *FGF20* on DPCs, we used DPCs transfected with FGF20‐siRNA‐1098, FGF20‐siRNA‐773, FGF20‐siRNA‐900, FGF20‐siRNA‐1006, siRNA‐NC or FAM‐labelled NC, and observed them under an inverted fluorescence microscope. More than 90% of the cells exhibited green fluorescence, indicating high transfection efficiency (Figure 4A). *FGF20* mRNA expression was measured by qRT‐PCR and was found to be significantly inhibited in DPCs transfected with FGF20‐siRNA‐1098 (*p* < 0.01, Figure 4B). Therefore, FGF20‐siRNA‐1098 was selected for subsequent experiments.

### 3.4 The Effect of *FGF20* Knockdown on the Expression Levels of *FGF20*‐Related Receptor Genes and Differentiation‐Related Pathway Marker Genes in HFSCs

**Figure 5:**
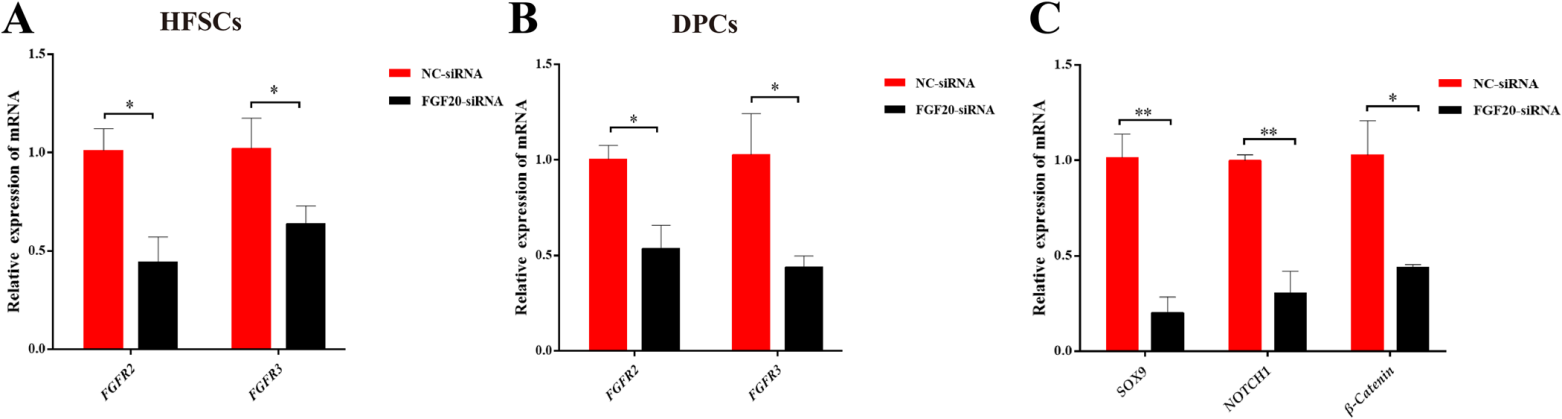
Detection of mRNA expression levels of *FGF20*‐related receptor genes and differentiation and pathway marker genes in co‐cultured cells by knocking down *FGF20* using qRT‐PCR. (A) The relative expression of *FGFR2* and *FGFR3* in HFSCs after DPCs treatment. (B) The relative expression of *FGFR2* and *FGFR3* in DPCs after DPCs treatment. (C) The effect of *FGF20* on the expression of HFSCs differentiation‐related genes after DPCs treatment. ** indicates *p* < 0.01, * indicates *p* < 0.05. [Color figure can be viewed at wileyonlinelibrary.com]

We used a co‐culture method to study the effect of *FGF20* knockdown on the expression levels of *FGF20*‐related receptor genes in cells, as well as on the expression levels of differentiation and pathway marker genes in HFSCs. Compared with the siRNA‐NC group, the FGF20‐siRNA group demonstrated a significant inhibition of mRNA expression for the *FGFR2* and *FGFR3* genes (*p* < 0.05) (Figure 5A,B). Similarly, the mRNA expression of differentiation and pathway marker genes, including *SOX9*, *NOTCH1* and *β‐Catenin*, was also inhibited (*p* < 0.05, Figure 5C), indicating that the knockdown of *FGF20* impeded the differentiation of HFSCs into hair follicles by suppressing Notch and Wnt signalling pathways.

### 3.5 EdU, CCK‐8 and Flow Cytometry Assay

**Figure 6:**
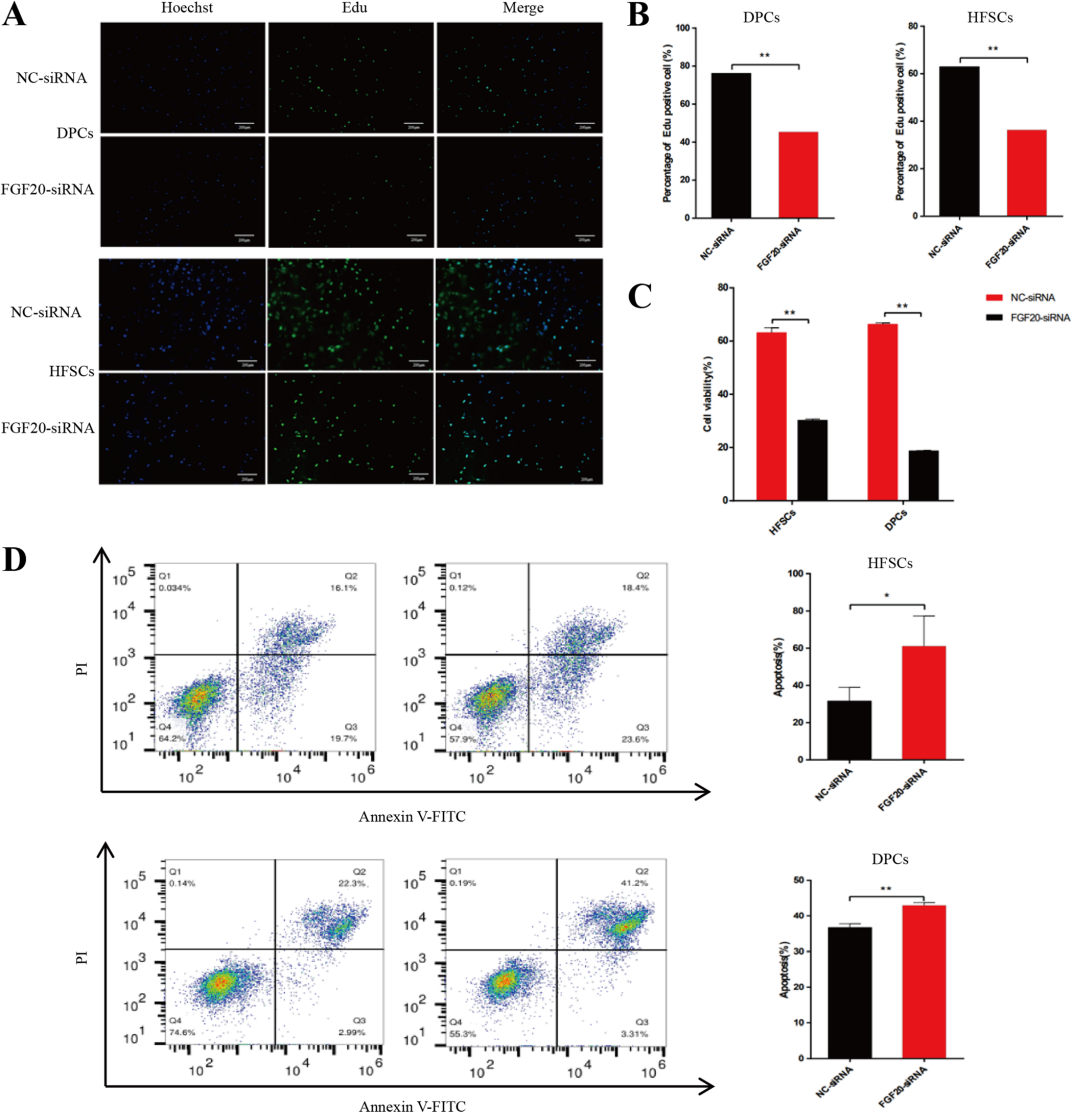
The effects of *FGF20* knockdown on the number of positive cells, cell viability and apoptosis in DPCs under co‐culture conditions were studied. (A) EdU‐stained HFSCs and DPCs were observed under an inverted fluorescence microscope; (B) The number of EdU‐positive cells in HFSCs and DPCs was counted. (C) The cell viability of HFSCs and DPCs was detected using CCK‐8. (D) The apoptosis rates of HFSCs and DPCs were detected by flow cytometry. ** indicates *p* < 0.01, * indicates *p* < 0.05. [Color figure can be viewed at wileyonlinelibrary.com]

To investigate the effect of *FGF20* on the proliferation and apoptosis of co‐cultured DPCs and HFSCs (P_4_ or P_5_), we transfected FGF20‐siRNA into the lower chamber of the co‐culture plate containing DPCs for 24 h, followed by the seeding of HFSCs in the upper chamber for an additional 24 h. Cell proliferation and apoptosis were detected using EdU, CCK‐8 and flow cytometry. The EdU results showed that positive cells could be observed under an inverted fluorescence microscope (Figure 6A), and the number of EdU‐positive cells in DPCs and HFSCs (Figure 6B) decreased significantly (*p* < 0.01). The CCK‐8 assay indicated a marked reduction in cell viability (*p* < 0.01, Figure 6C). Flow cytometry demonstrated that *FGF20* knockdown within the co‐culture system promoted apoptosis of HFSCs (*p* < 0.05) and DPCs (*p* < 0.01, Figure 6D). These results indicate that *FGF20*‐knockdown in DPCs inhibits the proliferation of DPCs and HFSCs.

## 4 Discussion

DPCs and HFSCs are the most important cells, and the interaction between them affects this regulation, such as the ratios and degrees of hair follicle morphogenesis (Ji et al. 2021). The establishment of optimized co‐cultured systems in vitro holds considerable significance for elucidating mechanisms underlying hair follicle morphogenesis (Williams, Profeta, and Stenn 1994). The reported isolation and culture methods of HFSCs include differential adhesion, microscopic separation, enzymatic digestion and tissue block culture (Garza et al. 2011; Choi et al. 2021; Yamane et al. 2021; Morris et al. 1990). These methods have the disadvantages of low cell separation efficiency, high costs, complicated operation, susceptibility to contamination and low purity. Zhang et al. (2006) isolated the bulge region of the hair follicle using neutral dispase, from which they obtained stem cells. Quan et al. (2016) obtained purified HFSCs by the Type IV collagenase adsorption method, which demonstrated excellent adsorption capacity and robust clone formation ability. In this study, DPCs were isolated through enzymatic digestion and mechanical separation. The isolated DPCs exhibited triangular or polygonal shapes and demonstrated distinct agglutination, reflecting their capacity for maintaining inductive properties, which aligns with prior findings reported by Bratka‐Robia et al. (2002). α‐SMA, a highly expressed transcription factor in DPCs, is mainly used to distinguish fibroblasts (Rendl, Lewis, and Fuchs 2005; Clavel et al. 2012), and it regulates the growth rate of hair follicles by regulating the BMP signalling pathway to act on the surrounding hair shaft precursor cells. CK14 (Inoue et al. 2009) and CK19 (Kloepper et al. 2008) are specific markers of HFSCs (Ma, Yang, and Lee 2004). In this study, the primary HFSCs of fine‐wool sheep obtained had a paving stone‐like morphology, which was consistent with a previous description (Hunt et al. 2008). Immunofluorescence was used to detect the expressions of α‐SMA and SOX2 in DPCs, and CK14 and CK19 in HFSCs, which were consistent with the results of reported studies (Rendl, Lewis, and Fuchs 2005; Inoue et al. 2009; Kloepper et al. 2008).

The wool fibre‐breaking elongation trait is an index of wool strength, which reflects the quality of wool (Long, Lei, and Guiqin 2008). The results of GWAS in this study showed that SNPs associated with the wool fibre‐breaking elongation trait were annotated to the *FGF20* gene. *FGF20* belongs to the *FGF* superfamily. Most members of it bind specifically to their fibroblast growth factor receptor (*FGFR*) and activate intracellular signalling pathways such as MAPK, PI3K‐AKT, and STAT in the cytoplasm, thereby regulating cell proliferation and differentiation (Zheng et al. 2010; Tezuka, Toyoshima, and Tsuji 2016; Su, Jin, and Chen 2014). In recent years, increasing attention has been focused on the study of *FGFs* in the development of certain neurological diseases, tumours, as well as in hair and teeth (Van der Walt et al. 2004; Ornitz and Itoh 2015; Beenken and Mohammadi 2009; Presta et al. 2017; Izaguirre et al. 2017). However, it has not been extensively studied in the hair follicle. The development and regulation of skin appendages such as hair are mainly based on the roles of the subfamilies *FGF7* and *FGF9*. *FGFR2* and *FGFR3* are receptors closely related to *FGF9*. *FGF20* is a member of the *FGF9* subfamily and has been shown to be associated with hair follicles (Huang, Xu, and Cheung 2017; Maiese 2015; Katoh 2008). After *FGF20* was knocked out by in situ hybridization, a partial loss of basal plate and dermal agglutination was observed in mouse hair follicles, which hindered the development of hair follicles (Huh et al. 2013). The absence of fibroblast growth factor receptor *FGFR2* reduces the number of epithelial cells, promotes hair follicle formation, and reduces the number of hair follicles (Ohuchi et al. 2000). Takenaka, Yasuno, and Kishimoto (2002) found that *FGFR3* is strongly expressed in pre‐epidermal cells. Related studies have shown that *FGF20* can specifically bind to tyrosine kinase receptors *FGFR2α* (*IIIb*), *FGFR2α* (*IIIc*), *FGFR2β* (*IIIb*) and *FGFR3α* (*IIIc*) on the cell surface through Wnt and BMP4 pathways, causing cell DNA synthesis and cell proliferation (Thisse and Thisse 2005; de Mena et al. 2010). In this study, the expression trends of *FGFR2* and *FGFR3* in DPCs and HGSCs after *FGF20* was knocked down were consistent. It was speculated that *FGF20* might be a ligand related to *FGFR2* and *FGFR3*. By binding to *FGFR2* and *FGFR3*, it promotes cell proliferation and differentiation, thereby participating in hair follicle formation.

Cellular interactions play an important regulatory role in proliferation and differentiation. Co‐culture models are established to simulate cellular interaction to study complex interactions between cells (Heydarkhan‐Hagvall et al. 2003; Khodarev et al. 2003; Bhatia, Yarmush, and Toner 1997; Orwin and Hubel 2000), which can incorporate different cell types into the same environment (Chi, Wu, and Morgan 2013; Higgins et al. 2013; Lin et al. 2014). At present, there are few reports on the co‐culture of DPCs and HFSCs. In this study, DPCs and HFSCs were successfully isolated from fine‐wool sheep, and the established co‐culture system provides an effective tool to study the cellular interaction during hair follicle morphogenesis. By inhibiting the expression of *FGF20* in DPCs of fine‐wool sheep and co‐culturing with HFSCs, we found that the cell viability and the number of EdU‐positive cells for HFSCs in the FGF20‐siRNA group were significantly lower than those in the NC siRNA group, and the number of apoptotic cells increased significantly. These data further indicate that *FGF20* knockdown in DPCs inhibits the proliferation of HFSCs in the co‐culture model.

*SOX9*, a member of the *SOX* gene family, can specifically bind to DNA sequences and play a key regulatory role in animal sex determination and chondrogenesis, plays an important role in maintaining the characteristics of early HFSCs and initiating cell proliferation and differentiation (Nowak et al. 2008). Greco et al. (2009) found that *SOX9* might be a specific marker for HFSCs. Wnt/β‐Catenin and Notch are classical pathways that promote the proliferation and differentiation of HFSCs and the major pathways that are first activated during hair regeneration (Bai et al. 2015). The differentiation direction of HFSCs is regulated by the expression of *β‐Catenin* (Huelsken and Birchmeier 2001). The Notch signalling pathway can promote the differentiation of HFSCs into hair follicle cells, inhibit their differentiation into epidermal cells and maintain the normal periodic growth of hair follicles. In this study, the results showed that knocking down *FGF20* in DPCs can inhibit the differentiation of HFSCs by inhibiting the Notch and Wnt/β‐Catenin signalling pathways, thus inhibiting hair follicle morphogenesis, providing a reference for further study of the effects on hair follicle development in different species.

## 5 Conclusion

In this study, primary DPCs and HFSCs of fine wool sheep were isolated, and an indirect coculture system was successfully established to simulate the microenvironment in vivo. In the coculture system, the knockdown of *FGF20* in DPCs inhibited the proliferation and cell viability of HFSCs and promoted cell apoptosis. The expression levels of differentiation and pathway marker genes, including *SOX9*, *NOTCH1* and *β‐Catenin*, were significantly downregulated in HFSCs, indicating that *FGF20* knockdown in DPCs inhibited the differentiation of HFSCs through Notch and Wnt/β‐Catenin signalling pathways. This also indicates that *FGF20* can promote the morphogenesis of hair follicles in fine‐wool sheep. This result can provide more theoretical bases for the study of fine‐wool sheep hair follicle development and also offer new ideas for accelerating the molecular breeding process of fine‐wool sheep.

## Author Contributions

**Tingting Guo:** conceptualization, funding acquisition. **Chao Yuan:** conceptualization, writing–review and editing. **Yuhang Li:** methodology, validation, formal analysis, data curation, writing–original draft preparation. **Tong Xiao:** software. **Zengkui Lu:** formal analysis. **Lin Yue:** data curation. **Jianbin Liu:** supervision. **Bohui Yang:** supervision. All authors have read and agreed to the published version of the manuscript.

## Ethics Statement

The authors confirm that the ethical policies of the journal, as noted on the journal's author guidelines page, have been adhered to and the appropriate ethical review committee approval has been received. The authors confirm that all animals were handled in strict accordance with good animal practice according to the Animal Ethics Procedures and Guidelines of the People's Republic of China, and the study was approved by The Animal Administration and Ethics Committee of Lanzhou Institute of Husbandry and Pharmaceutical Sciences of CAAS (Permit No. SYXK‐2014‐0002).

## Consent

The authors have nothing to report.

## Conflicts of Interest

The authors declare no conflicts of interest.

## Data Availability

The data used to support the findings of this study have not been made available as we will be conducting further research.
